# Para-Hisian atrial tachycardia: A neglected cause of severe heart failure in children

**DOI:** 10.21542/gcsp.2022.4

**Published:** 2022-06-30

**Authors:** Aliaa Tarek, Wessam Ali, Mohamed Sayed, Tarek Hammouda, Omnia Kamel

**Affiliations:** Aswan Heart Centre, Magdi Yacoub Foundation, Egypt

## Abstract

Incessant focal atrial tachycardia (FAT) is the most common cause of tachycardia-induced cardiomyopathy in pediatric patients and is usually a reversible condition with effective management of tachycardia, either with medical treatment or ablation. These patients may be misdiagnosed, potentially leading to inappropriate treatment. Diagnosis is often late and should always be suspected in patients with congestive heart failure and unexplained persistent tachycardia. Para-Hisian atrial tachycardia is not an uncommon type of FATs; however, catheter ablation of anterior atrial septum-ATs has been a challenge because of its proximity to the AV node and the complex anatomy of its region.

## Introduction

Focal atrial tachycardia originates from a circumscribed area with centrifugal spread to both atria. This could be due to enhanced automaticity, triggered activity, or micro-reentry. Sites of origin for focal AT tend to cluster at specific anatomical locations in the right and left atria, with crista terminalis being the most common location^1^. FAT is a common cause of chronic supraventricular tachycardia in children with an estimated prevalence of 0.34% to 0.46%^2,3^.

Despite representing a small percentage of the SVT forms, they account for the largest proportion of tachycardia-induced cardiomyopathy (TIC) encountered in children and infants^4^.

Medical therapy has been the primary treatment for FAT, especially in young children who are more likely to have spontaneous resolution^5,6^. Although a variety of antiarrhythmic medications have been effective, FAT is often resistant to pharmacological therapy^7,8^. Radiofrequency ablation (RFA) has been successfully used for the definitive management of arrhythmia and resolution of cardiomyopathy^9,10^. The use of three-dimensional electroanatomic mapping (3D EAM) during catheter ablation has improved the rates of acute and long-term control^11^.

Less commonly, symptomatic ATs arise from the superoparaseptal or para-Hisian regions. Mapping and ablation of these arrhythmias can pose unique challenges as sites of successful ablation may localize near the His bundle with small but significant risk of damage to the AV conduction system, therefore other approaches were adopted as ablation from left side of the atrial septum, or the non-coronary cusp (NCC) of the aortic sinus of Valsalva^3,4^.

We report a case of incessant para-Hisian atrial tachycardia-inducing cardiomyopathy in a pediatric patient with a review of the mapping and ablation approaches.

### Case

**Figure 1.**
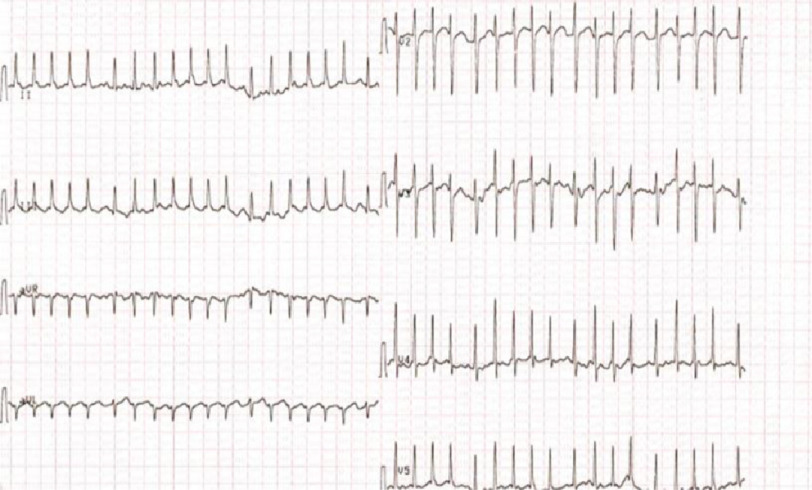
12-lead ECG shows long RP tachycardia.

An 11-year-old boy presented to our center with rapid palpitations associated with shortness of breath for 5 months without any history of cardiac conditions. His resting electrocardiogram (ECG) revealed narrow, complex, and long RP tachycardia (Figure 1).

**Figure 2.**
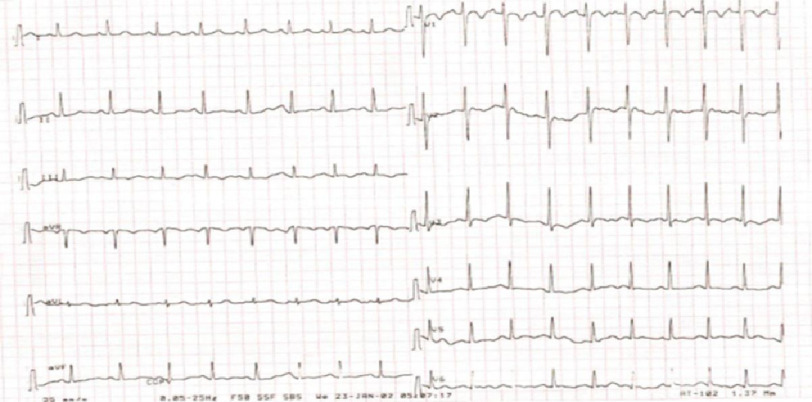
12-lead ECG after propranolol administration.

After giving an IV propranolol dose his heart rate was slowed down clarifying positive P wave in leads V1, II, III and aVF, suggesting FAT originating from RSPV or LAA (Figure 2).

**Figure 3.**
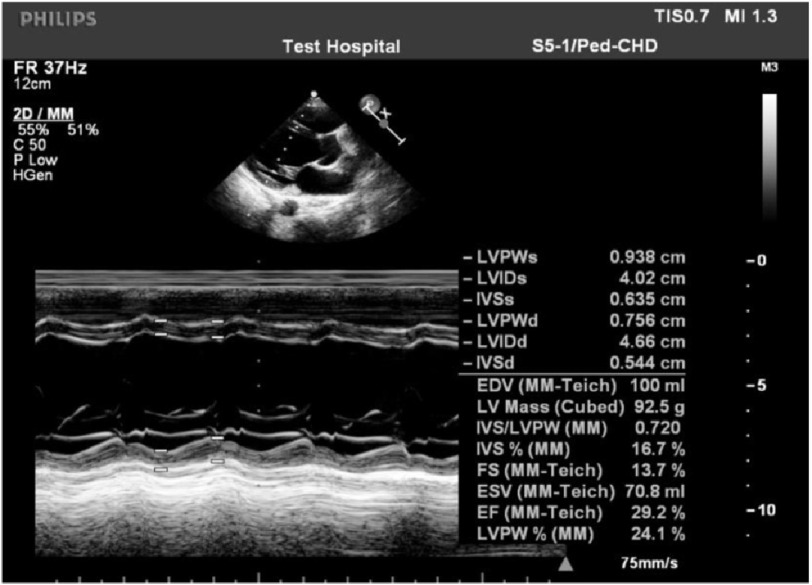
TTE, parasternal long-axis view: dilated LV dimensions and impaired LV systolic function.

Transthoracic echocardiography revealed dilated left ventricular (LV) dimensions and impaired systolic function (Figure 3).

Given the recurrent symptoms and patient reluctance to antiarrhythmic therapy in addition to impaired LV systolic function, electrophysiology studies and catheter ablation were performed.

**Figure 4.**
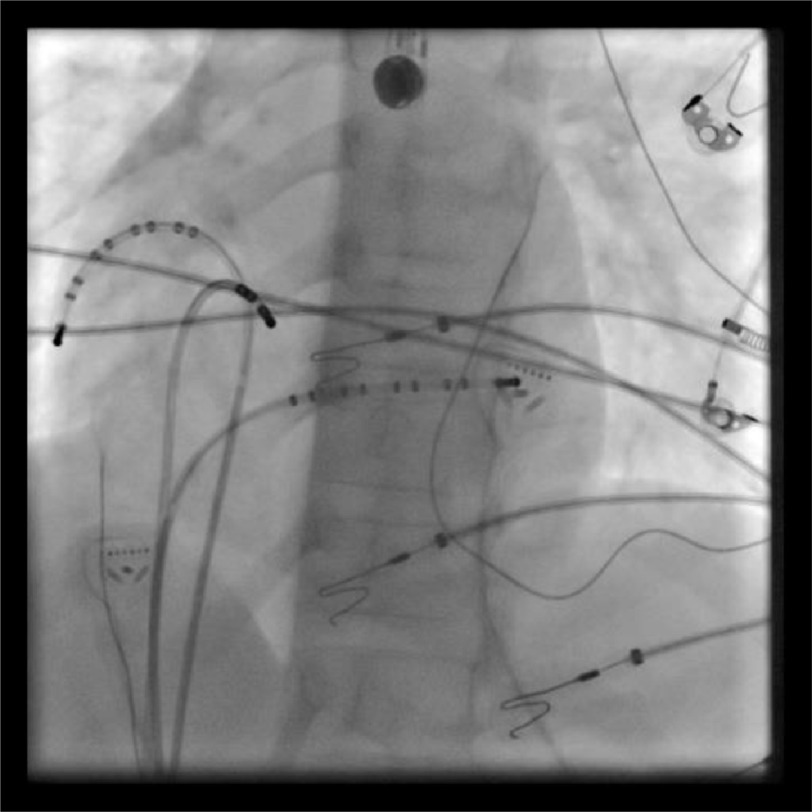
LAO view with two decapolar catheters in the RFW and CS, and one quadripolar catheter in the His bundle region.

**Figure 5.**
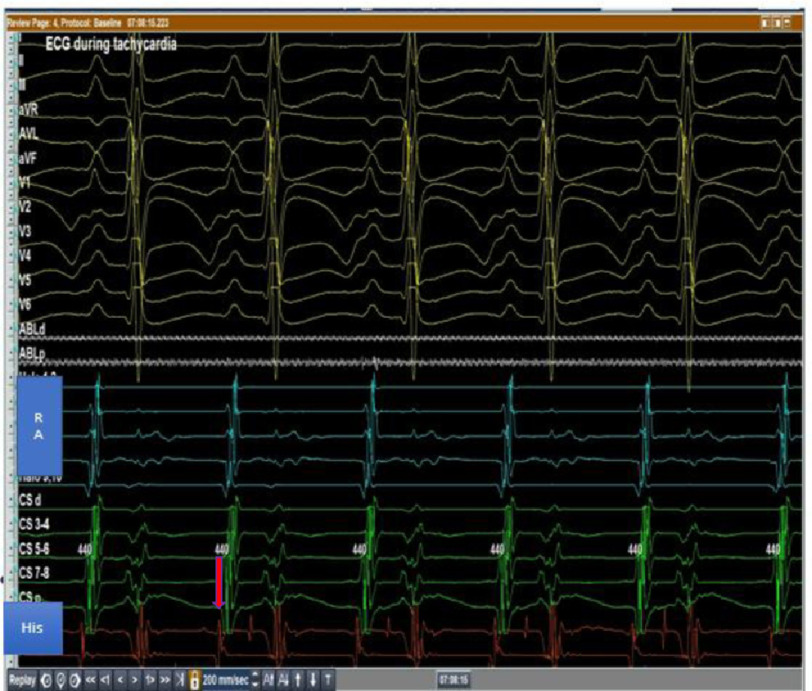
Intracardiac tracing of sustained tachycardia with long VA time, with earliest A signal at the proximal His catheter electrode.

The procedure was performed under general anesthesia after written consent was obtained from the parents. Multielectrode catheters were introduced via 7F sheath in right femoral vein, and two 6F sheaths in the left femoral vein. They were positioned in the right atrial free wall (RFW), coronary sinus (CS), and His bundle region under fluoroscopic guidance (Figure 4). Once the catheters were in place, they recorded sustained tachycardia with long VA time of 440 ms cycle length and earliest A wave was noted at the proximal His electrode (Figure 5).

After ventricular overdrive pacing with advancement of tachycardia to the pacing rate with VAAV response upon cessation of pacing, the diagnosis of atrial tachycardia was confirmed.

**Figure 6.**
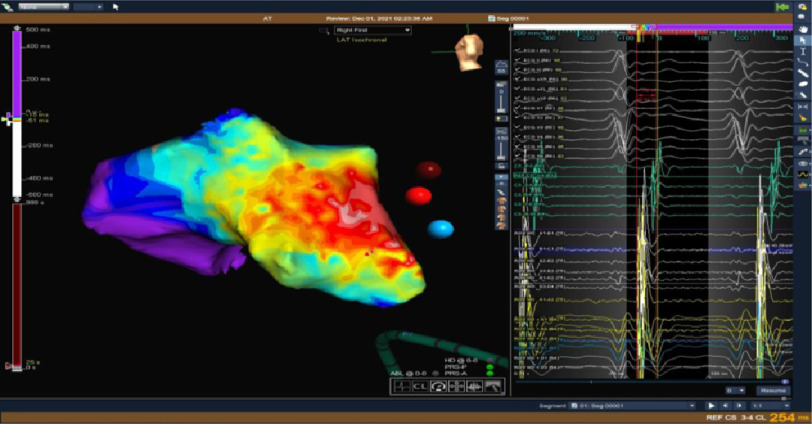
The activation map of the right atrium denotes the earliest activation in the para-Hisian region.

High density 3D EAM of the right atrium firstly was obtained detecting the earliest atrial activation signal at the para-Hisian region preceding the surface p wave onset by 42 ms with good unipolar signal (Figure 6).

Therefore, we decided to perform mapping of the left atrium. Transseptal puncture was performed (TEE and fluoroscopy-guided), with proper positioning of the long sheath in the left atrium under continuous heparinized saline infusion, IV heparin 100 IU per kg was given with a target ACT > 300 s.

**Figure 7.**
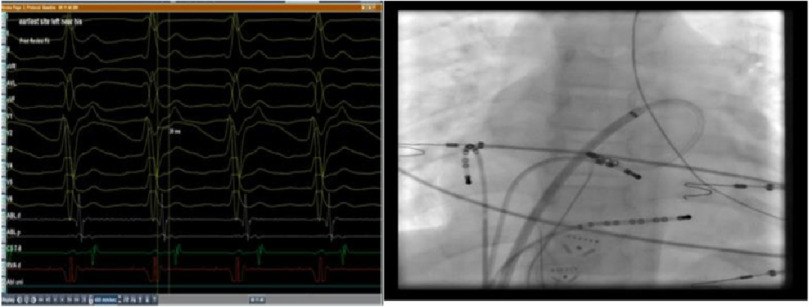
Intracardiac recording of the earliest activation of the left atrial septum preceded the surface P-wave by 34 ms and fluoroscopic LAO view of the ablation site at the left septum.

**Figure 8.**
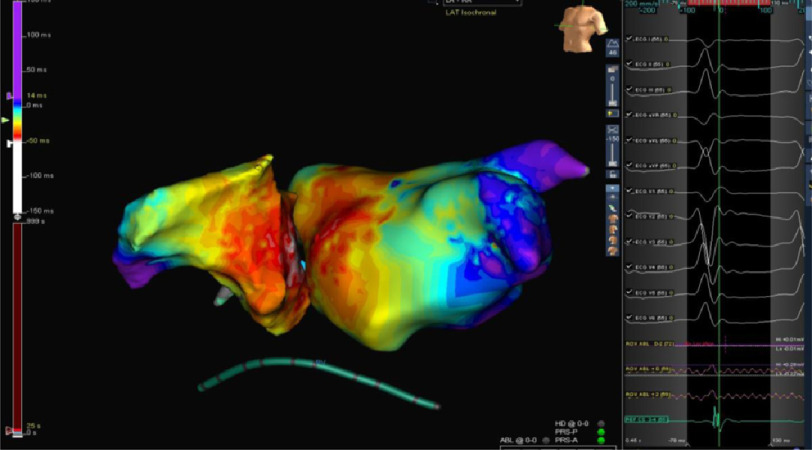
Activation map of both atria with para-Hisian area being the earliest.

However, mapping revealed the earliest atrial activation at the left septal area, which was later than that in the right para-Hisian region (Figure 7, Figure 8).

Given the higher risk of AV block from ablation from the right side, ablation from the left septal area seemed to be a good option. Thus, RF was applied using a contact force ablation catheter, but tachycardia was still sustained.

**Figure 9.**
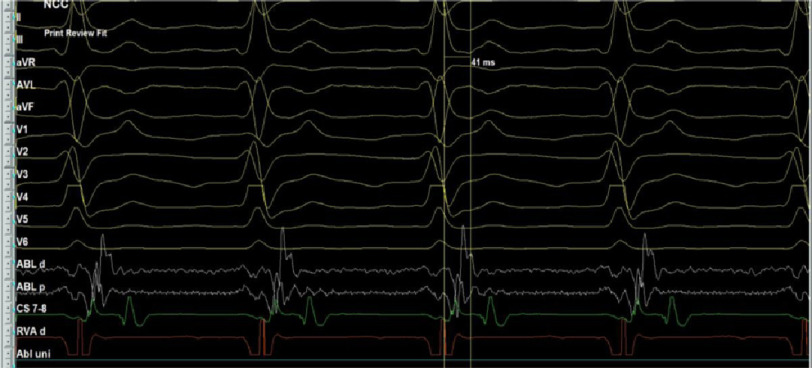
Intracardiac tracing of the early signals from the NCC.

Considering that the para-Hisian region was the earliest so far, mapping from NCC was an alternative for being in close anatomical proximity to the atrial septum and a much safer area in ablation. After obtaining right femoral arterial access, mapping was performed via a retrograde aortic approach recording an early signal preceding the surface P-wave by 41 ms (Figure 9), so RF energy was delivered for 60 s with a maximum of 35 watt and 55 degrees of temperature with adequate contact; however, tachycardia was not terminated.

**Figure 10.**
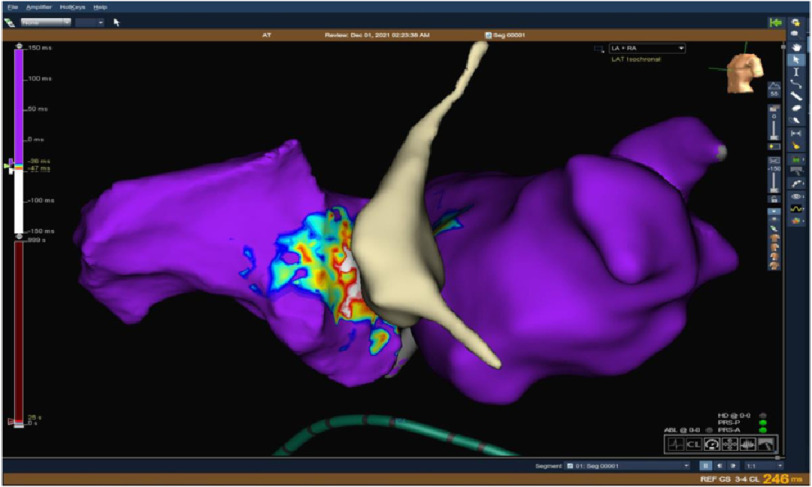
Activation map of both atria together with NCC confirming the para-Hisian origin of tachycardia.

At this level we were left with only one option with very little margin for error which was ablation from the right para-Hisian area (Figure 10).

**Figure 11.**
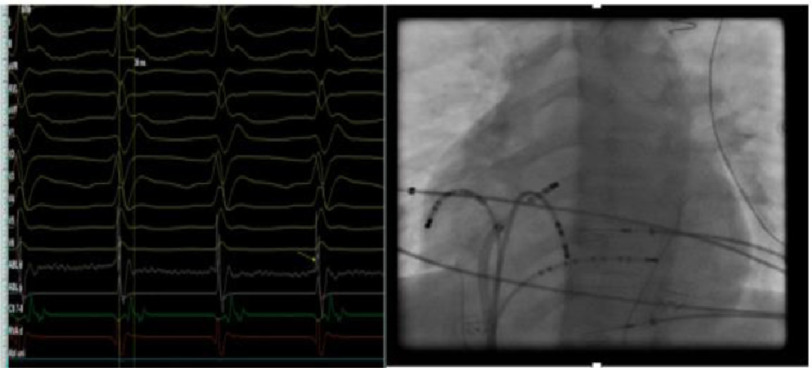
LAO view and intracardiac tracing of the site of successful ablation.

RFA was conducted starting at 15 W and titrated up to 25 W for 40 s with careful monitoring of AV time, with immediate termination of tachycardia followed by atrial pacing for catheter stability purpose (Figure 11–Figure 13). No further tachycardia induction despite giving maximum weight adjusted doses of isoprenaline.

**Figure 12.**
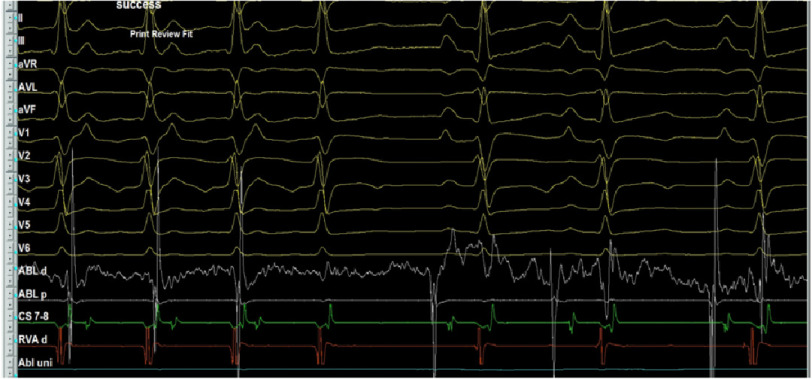
Immediate termination of tachycardia during ablation.

**Figure 13.**
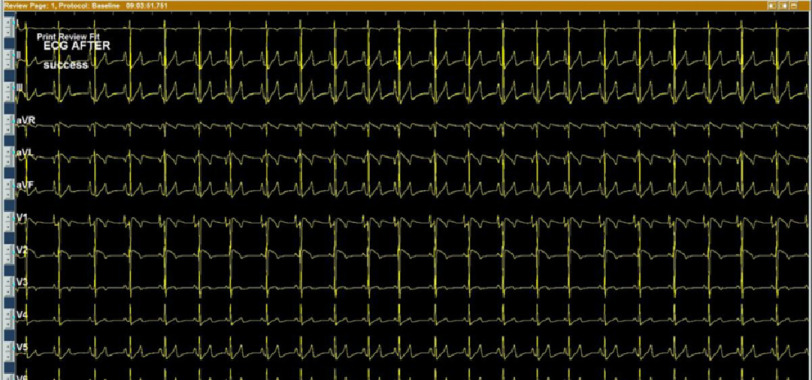
Post-ablation 12-lead ECG showing sinus rhythm.

**Figure 14.**
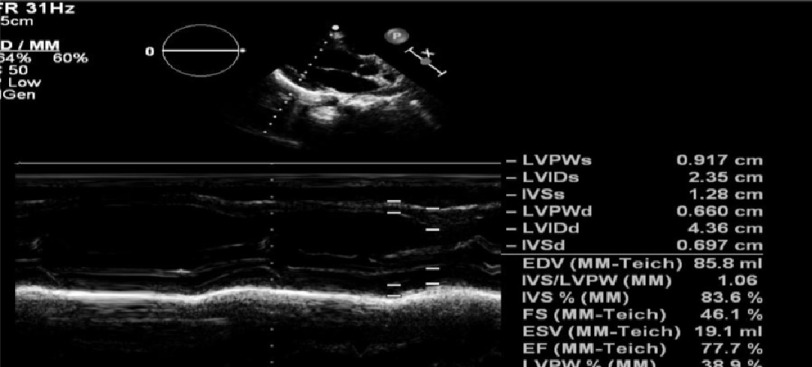
Parasternal long axis view: normal LV dimensions with normal systolic function.

The patient was discharged safely the day after the procedure. Three months later, the patient was in sinus rhythm with complete LV systolic function recovery (Figure 14).

## Discussion

FATs that arise near the atrioventricular (AV) node, so-called para-Hisian ATs, which are not uncommon in earlier studies, have challenged electrophysiologists to understand the mechanism of these arrhythmias and design safe and effective ablative strategies. The first descriptions of these tachycardias emphasized the importance of distinguishing them from AV nodal re-entry by EP diagnostic study^12,13^.

A number of algorithms using the P-wave morphology in the 12-lead ECG have been developed to determine the tachycardia origin; However, due to the complex anatomic relationship between both sides of the interatrial septum and the aortic root, the P-wave morphology does not accurately predict the successful ablation site and, therefore, detailed mapping of both atria together with aortic cusps may be needed in some instances^14^.

Tachycardia induced cardiomyopathy **(**TIC) is a ventricular dysfunction that occurs secondary to chronic and persistent tachycardia. Atrial tachycardia and permanent junctional reciprocating tachycardia are two frequent types of arrhythmias that can cause cardiomyopathy in children. This cardiomyopathy partially or totally improves with rhythm and heart rate control; therefore, early diagnosis is vital. In fact, the longer duration of tachycardia, the longer duration of remodeling.

Antiarrhythmic drugs class IA, IC, and III, as well as beta blockers, are used to treat these arrhythmias; however, in cases that are unresponsive to medical treatment, electrophysiological studies and radiofrequency catheter ablation are recommended^15^.

The obvious challenge in ablating para-Hisian ATs is to avoid collateral damage to the AV node, and this challenge becomes larger in pediatrics due to their small body surface area.

Subsequently, left atrial mapping was performed more frequently to identify arrhythmias best treated from the left side of the septum. Another approach is to use cryoablation in the right atrium (RA), which allows titration of cryoenergy to avoid permanent injury to the AV node. A breakthrough in approaching these ATs was the recognition that ablation in the NCC of the aortic valve can effectively treat these para-nodal ATs^16^.

Furthermore, it may appear paradoxical that ablation is successful from this site because there is no atrial myocardium per se in the aortic root^16,17^, but careful review of the anatomy in the para-septal region provides some clues that elucidate that the lowest part of the NCC is adjacent to the para-septal region and thus provides access to epicardial atrial tissue. It should also be emphasized that not all para-Hisian ATs can be ablated from the NCC, and some require ablation in the superoseptal RA, which could be performed effectively with extreme care to prevent inadvertent atrioventricular block by titrated energy application and continuous monitoring of atrioventricular conduction, as in our case. The best approach will ultimately depend on the distance and activation of a focal source from the AV node.

### Learning points

• Resting atrial tachycardia ECG in pediatrics could be a misleader in ablation, even in a structurally normal heart.
• Algorithms that looked up at P wave morphology have failed to show consistent ECG ‘signature’ for ATs arising from the para-Hisian region.
• Meticulous mapping of both atria is mandatory in these cases.
• NCC is not always helpful for para-Hisian atrial tachycardia.
• Attention to catheter position and titration of energy delivery is key in ensuring a safe outcome.
• Successful ablation of focal AT in those patients often restores normal left ventricular function.

## Abbreviations

FAT: Focal atrial tachycardia
TIC: Tachycardia induced cardiomyopathy
RFA: Radiofrequency ablation
3D EAM: Three dimensional electroanatomic mapping
RFW: Right free wall
AVN: Atrioventricular node
SVT: Supraventricular tachycardia
